# Organizational Climate, Ethical Work Environment, and Turnover Intentions Among Healthcare Workers in a Romanian Chronic Care Hospital: A Multidimensional Analysis

**DOI:** 10.3390/healthcare14020264

**Published:** 2026-01-21

**Authors:** Sorina Enășoni, Diana Szekely, Flavia Zara, Cristina Stefania Dumitru, Alexia Manole, Catalin Dumitru, Raul Patrascu, Alexandra Enache

**Affiliations:** 1 Doctoral School, “Victor Babes” University of Medicine and Pharmacy, E. Murgu Square, No. 2, 300041 Timisoara, Romania; 2 Department II of Microscopic Morphology, Discipline of Histology, “Victor Babes” University of Medicine and Pharmacy, E. Murgu Square, No. 2, 300041 Timisoara, Romania; 3 Faculty of Medicine and Pharmacy, University of Oradea, 410087 Oradea, Romania; 4 Department of Obstetrics and Gynecology, “Victor Babes” University of Medicine and Pharmacy, Eftimie Murgu Square 2, 300041 Timisoara, Romania; 5 Department of Functional Sciences, “Victor Babes” University of Medicine and Pharmacy, 300041 Timisoara, Romania; 6 Department VIII, Discipline of Forensic Medicine, Bioethics, Deontology and Medical Law, “Victor Babes” University of Medicine and Pharmacy, E. Murgu Square, Nr. 2, 300041 Timisoara, Romania; 7 Center for Ethics in Human Genetic Identifications, “Victor Babes” University of Medicine and Pharmacy, E. Murgu Square, Nr. 2, 300041 Timisoara, Romania

**Keywords:** health personnel, personnel turnover, job satisfaction, organizational culture, ethics, institutional, long-term care facilities

## Abstract

**Background:** Turnover intention among healthcare workers represents a growing challenge for chronic care institutions, particularly in resource-constrained healthcare systems. Organizational and ethical factors have been increasingly recognized as relevant correlates of workforce retention. **Methods:** This exploratory cross-sectional study was conducted in a Romanian chronic care hospital between January 2023 and September 2024. A total of 62 healthcare workers were included using a census-based recruitment approach. Organizational climate, ethical work environment, job satisfaction, and turnover intention were assessed using adapted and composite self-report measures. Correlation, multivariate regression, and exploratory mediation analyses were performed. **Results:** Job satisfaction and ethical work environment were inversely associated with turnover intention in both correlation and multivariate analyses. Organizational climate did not retain an independent association with turnover intention after adjustment. Exploratory mediation analysis suggested that job satisfaction may partially account for the association between an ethical work environment and turnover intention. Higher turnover intention and less favorable organizational perceptions were observed among nursing and auxiliary healthcare staff compared to physicians. **Conclusions:** The findings suggest that organizational and ethical conditions, particularly those influencing job satisfaction, are relevant correlates of turnover intention in chronic care settings. Given the exploratory design and limited sample size, these results should be interpreted cautiously. Organizational strategies targeting ethical practices and everyday work satisfaction may represent feasible approaches to workforce retention in similar healthcare contexts.

## 1. Introduction

Healthcare systems worldwide continue to face profound workforce challenges in the post-pandemic period, characterized by staff shortages, high turnover rates, and increasing difficulties in retaining experienced healthcare professionals. While much of the existing literature has emphasized individual-level psychological outcomes such as burnout, stress, and moral injury, recent evidence highlights that organizational-level factors—such as workplace culture, leadership, and systemic support—play a decisive role in shaping workforce sustainability and professional retention [1,2,3]. In this context, understanding how the organizational climate and ethical work environment influence healthcare workers’ intention to leave their job has become a critical priority for healthcare management and policy.

Turnover intention is widely recognized as one of the most robust predictors of actual workforce attrition and is linked to adverse consequences at both individual and system levels, including reduced continuity of care, increased organizational costs, and compromised patient safety [4,5]. Evidence shows that turnover intention among healthcare workers is not solely driven by workload or emotional exhaustion, but is strongly shaped by perceptions of organizational justice, leadership quality, ethical climate, and institutional support [6,7]. These insights have shifted the focus from resilience-based interventions targeting individuals toward organizational strategies that foster supportive environments in which healthcare professionals can thrive [8].

The concept of organizational climate encompasses shared perceptions of policies, practices, leadership behaviors, and interpersonal relationships within a workplace, shaping how employees experience their professional roles [9]. A positive organizational climate has been associated with higher job satisfaction, stronger organizational commitment, and lower turnover intention among healthcare workers [10,11]. Closely related, the ethical work environment reflects the extent to which ethical principles, fairness, transparency, and moral accountability are embedded in daily organizational practices and decision-making processes [12]. In healthcare settings, where professionals frequently face ethically complex situations, a deficient ethical climate may exacerbate moral tension, erode trust in leadership, and ultimately contribute to disengagement and intention to leave [6,13,14].

Importantly, these organizational and ethical constructs should not be viewed as independent or parallel determinants of turnover intention, but rather as interrelated components of a broader organizational mechanism [6,9]. Organizational climate represents the structural and managerial framework of the institution, shaping daily work processes, leadership practices, and communication patterns [9,11]. Within this framework, the ethical work environment reflects the extent to which fairness, transparency, and ethical principles are enacted in everyday organizational practices [12,13,14]. These dimensions are closely linked to job satisfaction, which functions as a more proximal determinant of healthcare workers’ intention to remain in or leave their organization [10,15,16,17]. Accordingly, organizational climate and ethical work environment were treated as analytically distinct constructs in this study, despite their conceptual relatedness, and were entered separately into the statistical models.

Previous research has consistently shown that organizational and ethical factors influence turnover intention both directly and indirectly, through mediating variables such as job satisfaction and organizational commitment [10,16,18]. In this sense, organizational climate may exert its influence primarily by shaping ethical perceptions and satisfaction with work conditions, which in turn affect turnover intention. Conceptualizing these relationships in a hierarchical rather than parallel manner allows for a more coherent analytical approach and provides a clearer rationale for multivariate and mediation analyses.

In addition to organizational and ethical determinants, turnover intention among healthcare workers has been shown to be influenced by individual and professional characteristics, including age, gender, professional role, years of experience, and hierarchical position within the organization [19]. These factors may act as confounders or moderators in the relationship between organizational conditions and turnover intention. Younger staff, nursing personnel, and auxiliary healthcare workers have been reported to exhibit higher turnover intention, particularly in resource-constrained healthcare settings [20,21,22]. Therefore, accounting for basic sociodemographic and professional attributes is essential for accurately estimating the independent contribution of organizational and ethical factors to turnover intention.

Chronic care hospitals represent a particularly vulnerable organizational context. These institutions are characterized by prolonged patient–provider relationships, sustained exposure to suffering and end-of-life care, limited visibility within healthcare systems, and often constrained material and human resources [15]. Such conditions intensify the importance of organizational and ethical factors, as healthcare workers must continuously navigate complex moral responsibilities within structurally demanding environments [13,16]. Despite this, chronic care settings remain underrepresented in empirical research on organizational climate and workforce retention, especially in Eastern European healthcare systems [22,23].

In Romania, public healthcare institutions—particularly chronic care hospitals—face long-standing structural challenges, including underfunding, workforce shortages, and limited access to organizational development resources [24]. Existing national and international studies have primarily examined burnout and stress-related outcomes among healthcare workers, while fewer investigations have addressed how organizational and ethical dimensions influence turnover intention in this specific context [25,26].

Building on this emerging evidence, the present study adopts an organizational-level perspective, moving beyond burnout-centered models to examine how organizational climate and ethical work environment relate to turnover intentions among healthcare workers. By focusing on a Romanian chronic care hospital as a single-center case study, this research aims to provide a nuanced, context-sensitive analysis of workforce dynamics in an underexplored healthcare sector. Such an approach aligns with contemporary calls to address healthcare workforce sustainability through structural and organizational interventions rather than solely individual-level coping strategies.

The primary objective of this study was to evaluate the associations between organizational climate, ethical work environment, and turnover intention among healthcare workers employed in a Romanian chronic care hospital. Secondary objectives included exploring differences across professional categories and examining the relative contribution of organizational and ethical factors to turnover intention within this institutional context.

Based on this conceptual framework, the present study examines organizational climate and ethical work environment as structural determinants of turnover intention, with job satisfaction considered as a proximal factor within this relationship, while accounting for key sociodemographic and professional characteristics. This approach provides the analytical foundation for the multivariate and exploratory mediation analyses presented in the subsequent sections.

## 2. Materials and Methods

### 2.1. Study Design and Setting

This study was designed as an observational, cross-sectional, single-center investigation, conducted in a Romanian chronic care hospital. The research was carried out at the Chronic Diseases Hospital of Sebiș, Arad County, Romania, a public long-term care institution providing continuous medical assistance to patients with chronic and degenerative conditions. The single-center design was deliberately chosen to allow for an in-depth, context-sensitive analysis of organizational and ethical factors within a chronic care environment, while ensuring methodological continuity with previous institutional research conducted in the same setting. Data collection was performed during the post-pandemic period, between January 2023 and September 2024, a timeframe characterized by persistent workforce strain and organizational restructuring in Romanian public healthcare institutions. Given the single-center design, the findings are not intended to be generalizable to all healthcare settings but rather to provide context-specific, exploratory insights into organizational and ethical factors associated with turnover intention.

### 2.2. Study Population and Participants

The study population consisted of healthcare workers employed at the Chronic Diseases Hospital of Sebiș during the study period. A total of 62 healthcare professionals were included in the analysis, reflecting the active clinical workforce of the institution at the time of data collection. During the study period, the active clinical workforce of the hospital comprised 62 eligible healthcare workers (physicians, nurses, and auxiliary staff) who met the inclusion criteria. All eligible employees were invited to participate (census-based recruitment), given the limited size of the institution and the exploratory single-center design. The sample comprised physicians, nurses, and auxiliary healthcare staff, representing the multidisciplinary structure typical of Romanian chronic care hospitals.

Inclusion criteria:

- active employment in the hospital during the study period;
- minimum of one year of professional experience within the institution;
- voluntary participation with provision of written informed consent.

Exclusion criteria:

- temporary absence from work due to medical or maternity leave during the data collection period;
- refusal or inability to complete the questionnaire.

Given the relatively small size of the institution and the limited number of eligible employees, this recruitment strategy was considered appropriate for exploratory organizational research in a single-center chronic care setting.

#### Sample Size Estimation and Statistical Power

Given the single-center design and the small workforce size of the institution, the study used a census-based recruitment approach (i.e., all eligible healthcare workers were invited to participate). Therefore, the achievable sample size was primarily constrained by the finite population of the hospital rather than by an unrestricted sampling frame.

To provide transparency regarding the adequacy of the final sample for the planned analyses, an a priori power analysis was conducted for the main multivariate model (multiple linear regression) using a conventional significance level (α = 0.05) and statistical power (1 − β = 0.80). The final regression model included six predictors (organizational climate, ethical work environment, job satisfaction, and the control variables age, years of experience, and professional role). Under these assumptions, a sample size of N = 104 would be required to detect a medium overall effect size (Cohen’s f^2^ = 0.15), whereas N = 52 would be sufficient to detect a large effect size (Cohen’s f^2^ = 0.35). The achieved sample (N = 62) therefore provided adequate power to detect large effects in the multivariable model, but it was underpowered for small-to-moderate effects; for this reason, multivariable and mediation findings were explicitly interpreted as exploratory.

For the bivariate association analyses, the achieved sample size (N = 62) provides approximately 80% power to detect correlation coefficients of r ≥ 0.35 (two-tailed α = 0.05), supporting the interpretation of moderate-to-strong associations.

### 2.3. Data Collection Procedure

Data were collected using anonymized, self-administered paper-based questionnaires, distributed by the research team during work breaks or after scheduled shifts. Participation was voluntary, and confidentiality was ensured by the absence of any identifying personal information on the questionnaires.

All completed questionnaires were subsequently digitized and prepared for statistical analysis. Data collection was conducted during the post-pandemic period (January 2023 and September 2024), aiming to capture persistent organizational and ethical perceptions that may continue to influence workforce attitudes and retention in long-term care institutions. In total, 62 questionnaires were distributed to eligible staff members, and 62 were returned. All returned questionnaires met completeness criteria and were included in the final analysis (valid questionnaires, *n* = 62). The overall response rate was therefore 100% (62/62), and the valid response rate was 100% (62/62). Questionnaire distribution was conducted in two administrative stages, corresponding to staff availability during the study period. In the first stage (January 2023), 34 questionnaires were distributed to eligible healthcare workers, all of which were returned and met validity criteria (response rate: 100%; valid response rate: 100%). In the second stage (September 2024), 28 questionnaires were distributed to eligible healthcare workers who met the inclusion criteria and had not participated in the earlier administrative stage. All 28 questionnaires were returned and were valid for analysis (response rate: 100%; valid response rate: 100%). The combined sample from both stages resulted in a final analytic sample of 62 participants.

### 2.4. Measures and Instruments

To achieve the objectives of the study, validated instruments and theory-informed adapted measures were used to assess organizational and ethical factors, job satisfaction, and turnover intention.

Organizational Climate. Organizational climate was assessed using an adapted version of the Organizational Climate Measure developed by Patterson et al. (2005) [27]. The adapted scale included items evaluating leadership support, communication quality, role clarity, and perceived organizational fairness, dimensions commonly examined in healthcare organizational research. Items were rated on a five-point Likert scale (1 = strongly disagree; 5 = strongly agree), with higher scores indicating a more favorable organizational climate.

Ethical Work Environment. The ethical work environment was assessed using a composite measure developed for this study, informed by the Ethical Climate Questionnaire originally proposed by Victor and Cullen (1988) [28] and subsequent adaptations in healthcare research. Item selection was guided by a review of the literature and focused on ethical leadership, transparency in decision-making, respect for professional values, and perceived fairness of institutional policies, particularly relevant in chronic care settings. The resulting eight-item scale was reviewed by members of the research team with expertise in healthcare ethics and organizational behavior to ensure content validity. Responses were recorded on a five-point Likert scale (1 = strongly disagree; 5 = strongly agree), with higher scores indicating a more supportive ethical work environment. Unlike established ethical climate typologies, this composite measure was designed to capture perceived ethical practices as enacted in everyday organizational interactions, rather than to classify the organization into predefined ethical climate categories.

Job Satisfaction. Job satisfaction was measured using a brief multi-item scale adapted from widely used job satisfaction instruments in healthcare research, including the Job Satisfaction Survey developed by Spector (1985) [29]. The scale consisted of five items assessing satisfaction with professional role, working conditions, workload, interpersonal relationships, and organizational support. Items were rated on a five-point Likert scale ranging from 1 (very dissatisfied) to 5 (very satisfied), with higher scores indicating greater overall job satisfaction.

Turnover Intention. Turnover intention was assessed using a standardized multi-item Turnover Intention Scale adapted from the model proposed by Mobley et al. (1978) [30]. The scale evaluated the frequency of thoughts about leaving the organization, intention to seek alternative employment, and perceived likelihood of leaving the institution in the near future. Higher scores reflected stronger turnover intentions.

All instruments were administered in Romanian. For scales without an available validated Romanian version, items were translated and culturally adapted by the research team using forward-translation procedures, with emphasis on semantic clarity and contextual relevance for chronic care practice.

The internal consistency of all multi-item scales was assessed using Cronbach’s alpha coefficient. In the present sample, organizational climate demonstrated good reliability (Cronbach’s α = 0.83), ethical work environment showed satisfactory internal consistency (Cronbach’s α = 0.75), job satisfaction demonstrated good reliability (Cronbach’s α = 0.81), and turnover intention showed high internal consistency (Cronbach’s α = 0.88). These values indicate acceptable to good reliability for all measures used in the study.

#### Conceptual Model and Analytical Framework

The analytical approach of this study was guided by a conceptual model grounded in organizational and ethical theories of workforce retention. In this framework, organizational climate and ethical work environment were conceptualized as distal organizational determinants influencing healthcare workers’ intention to leave their institution. Job satisfaction was positioned as a more proximal factor, reflecting healthcare workers’ day-to-day evaluation of their professional experience and serving as a potential pathway through which organizational and ethical conditions may affect turnover intention.

Turnover intention was defined as the primary outcome variable. Organizational climate and ethical work environment were treated as main explanatory variables, while job satisfaction was examined both as an independent predictor and as a potential mediator of the relationship between ethical and organizational factors and turnover intention. Key sociodemographic and professional characteristics (age, years of experience, and professional role) were included as control variables to account for their previously documented influence on turnover intention.

This conceptual model informed the selection of variables for the multivariate regression analyses and provided the rationale for the exploratory mediation analysis performed in this study.

### 2.5. Statistical Analysis

Statistical analyses were performed using MedCalc (version 22.0). Descriptive statistics were used to summarize sociodemographic and professional characteristics. Given the finite population size of the institution and the exploratory aim of this single-center study, power considerations are reported in Section Sample Size Estimation and Statistical Power, and multivariable and mediation analyses were interpreted cautiously. Continuous variables were expressed as means ± standard deviations, while categorical variables were presented as frequencies and percentages. Bivariate associations between organizational climate, ethical work environment, job satisfaction, and turnover intention were examined using Pearson or Spearman correlation coefficients, as appropriate. Group differences across professional categories were assessed using independent samples *t*-tests or one-way analysis of variance (ANOVA).

To identify independent predictors of turnover intention, multivariate linear regression models were constructed, incorporating organizational climate, ethical work environment, and job satisfaction as explanatory variables, while adjusting for age, professional role, and years of experience. In exploratory analyses, mediation models were examined to assess whether job satisfaction mediated the relationship between organizational or ethical factors and turnover intention.

The threshold for statistical significance was set at *p* < 0.05. Given the exploratory nature of the study and the single-center design, results were interpreted with appropriate caution regarding external generalizability. Given the limited sample size and cross-sectional design, all regression and mediation analyses were conducted with an exploratory aim and interpreted as hypothesis-generating rather than confirmatory.

## 3. Results

### 3.1. Participant Characteristics

A total of 62 healthcare workers from the Chronic Diseases Hospital of Sebiș were included in the final analysis. The sample comprised 6 physicians (9.7%), 34 nurses (54.8%), and 22 auxiliary healthcare staff members (35.5%), reflecting the actual multidisciplinary structure of the institution. The response rate was 100%, and all returned questionnaires were valid for analysis.

The mean age of participants was 40.2 ± 9.6 years (range: 25–62 years), and the majority of respondents were female (75.0%). Professional experience ranged from 1 to 35 years, with a mean duration of 12.3 ± 8.7 years. No statistically significant differences in age or years of experience were observed across professional categories (*p* > 0.05).

### 3.2. Descriptive Statistics of Organizational and Work-Related Variables

Descriptive statistics for the main study variables are summarized in Table 1. Overall, participants reported moderate levels of organizational climate and ethical work environment, alongside moderate job satisfaction and notable turnover intention.

The mean organizational climate score was 3.21 ± 0.54, suggesting a moderately positive perception of leadership support, communication, and organizational fairness. The ethical work environment score averaged 3.34 ± 0.61, indicating a generally acceptable but not strongly supportive ethical climate within the institution.

Job satisfaction scores were moderate (3.18 ± 0.66), while turnover intention demonstrated a relatively elevated mean value (3.01 ± 0.72), reflecting a substantial proportion of healthcare workers considering leaving the institution.

### 3.3. Differences Across Professional Categories

Comparative analyses revealed significant differences across professional roles for several key variables. Nurses and auxiliary healthcare staff reported lower organizational climate and ethical work environment scores compared to physicians (*p* < 0.05). Similarly, job satisfaction was significantly higher among physicians than among nurses and auxiliary staff (*p* = 0.031).

Turnover intention was highest among auxiliary healthcare staff (mean = 3.26 ± 0.68), followed by nurses (3.05 ± 0.71), and lowest among physicians (2.42 ± 0.59). These differences reached statistical significance (*p* = 0.018), suggesting that frontline and support staff may be particularly vulnerable to disengagement and workforce instability in the chronic care setting.

### 3.4. Correlation Analysis

Pearson correlation analysis was conducted to explore associations between organizational climate, ethical work environment, job satisfaction, and turnover intention. The results of the correlation analysis are presented in Table 2.

Organizational climate showed a moderate positive correlation with job satisfaction (r = 0.46, *p* < 0.001) and a moderate negative correlation with turnover intention (r = −0.42, *p* = 0.001). Similarly, an ethical work environment was positively associated with job satisfaction (r = 0.51, *p* < 0.001) and negatively associated with turnover intention (r = −0.48, *p* < 0.001).

Job satisfaction demonstrated the strongest inverse relationship with turnover intention (r = −0.57, *p* < 0.001), indicating that lower satisfaction levels were closely linked to increased intention to leave the institution. Given the sample size, these effect estimates should be interpreted cautiously.

### 3.5. Multivariate Analysis of Turnover Intention

To identify independent predictors of turnover intention, a multivariate linear regression model was constructed, including organizational climate, ethical work environment, and job satisfaction as explanatory variables, adjusted for age, professional role, and years of experience. The results are summarized in Table 3.

In the adjusted model, job satisfaction emerged as the strongest independent predictor of turnover intention (β = −0.41, *p* = 0.002). Ethical work environment also remained a significant predictor (β = −0.29, *p* = 0.014), whereas organizational climate showed a weaker, non-significant effect after adjustment (β = −0.17, *p* = 0.087).

The overall model explained approximately 38% of the variance in turnover intention (adjusted R^2^ = 0.38), indicating a substantial contribution of organizational and ethical factors to workforce retention in this chronic care setting.

The multivariate regression results are summarized in Table 3, which reports standardized effect estimates, confidence intervals, and model fit statistics. Job satisfaction exhibited the strongest independent negative association with turnover intention. An ethical work environment also contributed significantly to the model, whereas the direct effect of organizational climate did not retain statistical significance after adjustment for relevant covariates. Given the limited sample size and cross-sectional design, all reported effect estimates should be interpreted cautiously and are presented for exploratory, hypothesis-generating purposes only.

### 3.6. Exploratory Mediation Analysis

Given the exploratory nature of the study and the limited sample size, an exploratory mediation analysis was conducted to examine whether job satisfaction may partially account for the association between ethical work environment and turnover intention. The analysis suggested a pattern of partial mediation, with job satisfaction accounting for approximately 45% of the total association between ethical work environment and turnover intention. Specifically, an ethical work environment was significantly associated with job satisfaction (β = −0.29, *p* = 0.014), and job satisfaction was significantly associated with turnover intention (β = −0.41, *p* = 0.002). When job satisfaction was included in the model, the direct effect of ethical work environment on turnover intention was attenuated but remained statistically significant, supporting a pattern of partial mediation.

## 4. Discussion

This exploratory single-center study examined the associations between organizational climate, ethical work environment, job satisfaction, and turnover intention among healthcare workers in a Romanian chronic care hospital. The findings suggest that turnover intention in this setting is associated with organizational and ethical perceptions, with job satisfaction and ethical work environment emerging as the most relevant correlates. Overall, the results indicate that healthcare workers in this chronic care hospital reported moderate organizational and ethical perceptions, accompanied by moderate job satisfaction and relatively elevated turnover intention. Correlation and multivariate analyses consistently showed that job satisfaction and ethical work environment were inversely associated with turnover intention, while organizational climate did not retain an independent association after adjustment [6,16]. This pattern suggests that broader climate perceptions may influence turnover intention primarily through more proximal factors, such as ethical practices and day-to-day work satisfaction. These findings are broadly consistent with previous research emphasizing the importance of organizational and ethical conditions for workforce retention in healthcare settings [17,18,19]. The lack of an independent effect of organizational climate after adjustment should not be interpreted as evidence of irrelevance. Rather, it may indicate overlapping variance with ethical work environment and job satisfaction, and the possibility that broader climate perceptions influence turnover intention primarily through these more immediate mediators [16]. From an organizational perspective, this pattern is meaningful: interventions focused solely on general workplace climate may have limited impact unless they translate into tangible ethical practices and improvements in staff satisfaction [14,18,31].

Differences across professional categories were observed, with nurses and auxiliary healthcare staff reporting lower organizational and ethical perceptions and higher turnover intention compared to physicians. These findings suggest that frontline and support staff may be particularly vulnerable to disengagement in chronic care settings, especially in organizational contexts characterized by sustained emotional demands and limited professional autonomy [32,33,34,35]. However, given the limited sample size and the small number of physicians included, these role-specific differences should be interpreted cautiously. Importantly, these findings complement previous research conducted in similar institutional contexts, where burnout and moral injury did not fully account for healthcare workers’ intentions to leave. Taken together, this body of evidence suggests that emotional exhaustion and moral distress may be downstream consequences of broader organizational and ethical deficiencies. As such, interventions aimed solely at enhancing individual resilience or coping strategies may be insufficient unless accompanied by structural changes that strengthen ethical leadership, transparency, and institutional support.

The findings of this study have important practical implications for healthcare management, particularly in chronic care institutions where workforce stability is critical for continuity and quality of care. The strong association between job satisfaction, ethical work environment, and turnover intention suggests that retention strategies should extend beyond workload redistribution or financial incentives and focus on strengthening organizational and ethical infrastructures.

From an organizational perspective, interventions aimed at improving ethical leadership, transparency in decision-making, and perceived fairness may have a direct impact on workforce retention [18,36,37]. Establishing clear ethical guidelines, encouraging open communication between staff and management, and fostering inclusive decision-making processes may enhance trust in leadership and reduce disengagement [38,39,40]. Importantly, such measures may be particularly beneficial for nurses and auxiliary healthcare staff, who appear more vulnerable to turnover intention [14,41].

The prominence of job satisfaction as a predictor of turnover intention underscores the importance of addressing everyday working conditions. Opportunities for professional development, recognition of professional contribution, and supportive supervisory relationships may represent feasible and effective strategies for improving satisfaction levels [17,42]. In chronic care settings, where emotional demands are sustained and outcomes are often less immediately rewarding, reinforcing professional meaning and institutional support may be especially relevant [35,41].

Chronic care hospitals in Romania operate within a healthcare system marked by persistent workforce shortages, limited resources, and increasing demands on healthcare professionals [22,43]. In this context, understanding the organizational and ethical determinants of turnover intention is essential for developing sustainable retention policies [6,16].

The present study provides context-specific evidence that ethical and organizational dimensions play a substantial role in shaping workforce attitudes in Romanian chronic care institutions. Unlike acute care settings, where workload intensity and time pressure may dominate, chronic care environments are characterized by prolonged patient interaction and cumulative emotional exposure [43]. This distinction reinforces the importance of ethical climate and organizational support as determinants of long-term professional engagement [6].

By offering empirical data from an underrepresented healthcare sector, this study contributes to the growing recognition that retention strategies must be tailored to the institutional context. Policymakers and healthcare administrators may benefit from incorporating organizational ethics and job satisfaction metrics into routine workforce assessments, thereby enabling early identification of disengagement and targeted intervention [17].

The findings of this study must be interpreted in light of several important limitations. First, the single-center design and relatively small sample size limit the generalizability of the results. In particular, the small number of physicians included warrants caution when interpreting role-specific comparisons. Second, the cross-sectional nature of the study precludes causal inference, and the observed associations should be interpreted as correlational. Additionally, the use of self-reported measures may introduce response bias, although questionnaire anonymity and the consistency of findings across analytical approaches partially mitigate this concern.

Future research should aim to replicate these findings in larger, multi-center samples and explore longitudinal designs to better capture causal relationships between organizational and ethical factors and workforce retention. Further studies may also examine the interaction between organizational variables and individual psychological outcomes, such as burnout or moral distress, to clarify their relative and combined contributions to turnover intention.

## 5. Conclusions

This study demonstrates that turnover intention among healthcare workers in a Romanian chronic care hospital is strongly associated with organizational and ethical factors, particularly job satisfaction and ethical work environment. The findings suggest that organizational climate influences workforce retention primarily through these more proximal determinants. Addressing ethical leadership, institutional fairness, and everyday working conditions may therefore represent key strategies for improving workforce stability in chronic care settings.

## Figures and Tables

**Table 1:** Descriptive statistics are presented as mean ± standard deviation (SD). Scores were calculated as average values across scale items. Higher scores indicate a more favorable organizational climate, ethical work environment, and job satisfaction, and higher turnover intention, respectively.

Variable	Mean ± SD	Minimum	Maximum
Organizational climate (1–5)	3.21 ± 0.54	2.10	4.40
Ethical work environment (1–5)	3.34 ± 0.61	2.00	4.60
Job satisfaction (1–5)	3.18 ± 0.66	1.80	4.60
Turnover intention (1–5)	3.01 ± 0.72	1.60	4.80

**Table 2:** Pearson correlation coefficients between organizational climate, ethical work environment, job satisfaction, and turnover intention. * *p* < 0.05.

Variable	Organizational Climate	Ethical Work Environment	Job Satisfaction	Turnover Intention
Organizational climate	1			
Ethical work environment	0.48 *	1		
Job satisfaction	0.46 *	0.51 *	1	
Turnover intention	−0.42 *	−0.48 *	−0.57 *	1

**Table 3:** Multivariate linear regression analysis was performed to identify independent predictors of turnover intention. Turnover intention was entered as the dependent variable. The model included organizational climate, ethical work environment, and job satisfaction as main explanatory variables and was adjusted for age, years of professional experience, and professional role. Standardized beta coefficients (β), standard errors (SE), 95% confidence intervals (CI), and *p*-values are reported. Model fit was assessed using the F-test and adjusted coefficient of determination (Adjusted R^2^). Statistical significance was set at *p* < 0.05.

Predictor Variable	β (Standardized)	SE	95% CI	*p*-Value
Organizational climate	−0.17	0.09	−0.36 to 0.02	0.087
Ethical work environment	−0.29	0.11	−0.51 to −0.06	0.014 *
Job satisfaction	−0.41	0.12	−0.65 to −0.18	0.002 *
Age (years)	−0.08	0.07	−0.21 to 0.06	0.251
Years of experience	−0.05	0.06	−0.17 to 0.08	0.436
Professional role	0.12	0.08	−0.04 to 0.29	0.138

Model statistics: Adjusted R^2^ = 0.38; F(6,55) = 6.72; *p* < 0.001. * Statistically significant at *p* < 0.05.

## Data Availability

The data presented in this study are available from the corresponding author upon reasonable request. The data are not publicly available due to ethical restrictions and the inclusion of potentially identifiable information related to a small sample of healthcare workers from a single institution.
